# A 20-year evidence-based experience of the evolving medicine regulation in Zanzibar

**DOI:** 10.1080/20523211.2024.2421273

**Published:** 2024-11-07

**Authors:** Burhani Simai, Daniel Joshua, Salma Ali, Bora Lichanda, Sharifa Ali, Amne Issa, Heber Anandan, Raphael Zozimus Sangeda

**Affiliations:** a Department of Medicine and Cosmetics, Zanzibar Food and Drug Agency, Zanzibar, Tanzania; b Department of Human Research, Zanzibar Health Research Institute, Zanzibar, Tanzania; c Department of Clinical Research, Dr. Agarwal's Health Care Ltd, Tirunelveli, South India; d Department of Pharmaceutical Microbiology, Muhimbili University of Health and Allied Sciences, Dar es Salaam, Tanzania

**Keywords:** Medicine regulation, Zanzibar, regulatory framework, public health, pharmaceutical quality, Tanzania

## Abstract

**Background:**

Access to medicine is crucial for the effective functioning of healthcare systems. A robust regulatory framework is necessary to ensure the safety, effectiveness, and availability. However, weak regulatory frameworks persist in many low- and middle-income countries, leasing to the circulation of falsified and substandard medicines as well as anti-competitive restrictions on registering poor-quality medicines, which poses a significant public health threat. This study evaluated the evolution of Zanzibar’s medicine regulatory system over the past two decades and identified the key factors contributing to its success by elaborating on the Zanzibar Food and Drug Agency, seeking the expertise of regional, continental, and global experts to assess its regulatory capacity and maturity level.

**Methods:**

This study was conducted at the Zanzibar Food and Drug Agency (ZFDA) in Unguja, Zanzibar, using a retrospective cross-sectional review and qualitative approach. It thoroughly reviewed relevant regulatory documents, including Acts, policies, guidelines, and assessment reports. Data were collected using a standardised checklist and analysed to uncover patterns and insights regarding the evolution of Zanzibar’s medicine regulatory system.

**Results:**

This study revealed substantial legal provisions, organisational development, strategic planning, and resource allocation improvements. Notable achievements include establishing a structured organisational framework, developing a comprehensive strategic plan, and implementing a Quality Management System (ISO 9001:2015 certified). The ZFDA also addressed human resource limitations by creating job descriptions and a staff scheme of service, enhancing financial resources through revised fee regulations and government support, and improving infrastructure with new office and laboratory facilities.

**Conclusion:**

Zanzibar’s medicine regulations have evolved significantly, with marked regulatory capacity and infrastructure improvements. Future efforts should address the remaining challenges and foster collaboration with regional and international bodies to ensure the continued evolution and effectiveness of Zanzibar’s medicine regulatory framework.

## Introduction

In any country, a robust regulatory framework is essential to ensure that accessible medicines are of high quality, safe, and effective. These regulatory frameworks vary significantly (Rägo et al., 2014). While some countries have well-established and efficient systems, others have encountered considerable challenges. An effective regulatory system requires well-developed and implemented policies, guidelines, and standards (Pejović et al., 2016). However, many low- and middle-income countries (LMICs) have struggled to establish and maintain effective medicine regulatory systems.

The challenges include limited resources, inadequate capacity and expertise, political instability and corruption (Roth et al., 2018). Consequently, poorly functioning medicine systems lack access to safe and effective medicines, which poses a significant public health threat, especially for medicines for priority diseases, such as infectious diseases (Bassat et al., 2016; Wada et al., 2022). Furthermore, these challenges have been linked to the widespread availability of substandard and falsified medicines, which contribute to major pharmaceutical crimes (Bassat et al., 2016).

Despite setbacks faced by LMICs, several successful initiatives have aimed to transform the medicine regulatory systems in these regions. For instance, the East African Community's Medicines Regulatory Harmonization (EAC MRH) (Narsai et al., 2012; Ndomondo-Sigonda & Ambali, 2011) and African Medicines Regulatory Harmonization (AMRH) have been established on the African continent. These initiatives have been instrumental in promoting the harmonisation of medicine regulatory systems across the region and continent, as well as in strengthening the capacity of regulatory authorities within individual countries.

Zanzibar, an archipelago off the coast of East Africa, is a semi-autonomous part of the United Republic of Tanzania (URT), with its own government structure, responsible for all non-union matters. While Zanzibar and mainland Tanzania share a currency and have integrated economies, certain sectors, including health, are not union matters and are governed independently. Consequently, the Zanzibar Food and Drugs Agency (ZFDA) and the Tanzania Medicines and Medical Devices Authority (TMDA) operate as two distinct regulatory authorities. There is a mutual understanding and collaboration between the two National Regulatory Authorities (NRAs) to ensure cohesive and effective regulation in the URT. The TMDA has achieved WHO maturity level 3, indicating a stable and well-functioning regulatory system. On the other hand, the ZFDA is at WHO maturity level 1, as per the 2022 on-site WHO benchmarking of Zanzibar regulatory system. Despite the differences in maturity levels, the existence of separate regulatory bodies is warranted due to the distinct administrative and governance structures in Zanzibar and mainland Tanzania. The island has undergone a significant transformation in regulatory affairs over the past 20 years. Before this transformation, the medicine regulatory environment on the island was characterised by a lack of transparency, accountability, and regulatory oversight. The situation was further exacerbated by factors such as weak health systems and limited human and financial resources (Broojerdi et al., 2020). To address these challenges, Zanzibar, through the Ministry of Health (MoH), proposed establishing a Medicine Regulatory Board, which, among other functions, shall be responsible for governing medicine regulation, developing and providing guidance documents to all stakeholders, and enhancing the skills and capacity of the medicine regulatory staff in Zanzibar. The Zanzibar House of Representatives approved the Board through the Zanzibar Food, Drugs, and Cosmetics (ZFDC) Act No. 2/2006 as the Zanzibar Food and Drugs Board and its Amendment Act No. 3/2017, which provided for the establishment of the Zanzibar Food and Drug Agency (ZFDA) (World Health Organization, 2016). Understanding the rationale behind Zanzibar’s pursuit of a resilient and effective regulatory framework will contribute to broader discussions on strengthening health systems in resource-limited settings. The study aimed to evaluate the evolution of Zanzibar’s medicine regulatory affairs over the past 20 years, with a specific focus on identifying the key factors that have contributed to its success.

## Methods

### Study setting

This study was conducted at the Zanzibar Food and Drug Agency (ZFDA) office in Unguja, Zanzibar. The office is in Mombasa, Changu Road, Zanzibar (Zanzibar Food and Drug Agency, 2024). Initially established as the Pharmacy Board in 1986 by the Pharmaceutical and Dangerous Drugs Act No. 2 of 1986, it was then transformed into the Zanzibar Food and Drugs Board (ZFDB) in 2006 (according to ZFDC Act No. 2 of 2006). Finaly, in 2017, it was established as a government agency, the ZFDA, under the Zanzibar Food, Drugs and Cosmetics (Amendment) Act, No. 3 of 2017 (ZFDA, 2013).

## Study design and period

This study employed a cross-sectional design and qualitative approach. The study design employed three phases: desk review, data analysis, and compilation of the findings. This approach thoroughly reviewed relevant regulatory documents, including Acts, Regulations, Policies, guidelines, standard operating procedures (SOPs), and assessment reports. This design enabled for data collection at specific time points during the study. Furthermore, the research team comprised local and international academics as well as employees of the agency, ensuring a balanced perspective and enhancing the objectivity of the analysis. All researchers signed confidentiality and non-disclosure agreements before being granted access to information not in the public domain, including some confidential documentation. This ensured that all data used in the study was appropriately accessed and legally obtained.

## Data sources

This study involved several regulatory documents that evaluated the evolution of Zanzibar’s medicine regulatory affairs over the past 20 years. The regulatory documents reviewed included ZFDC Act No. 2 of 2006 and its amendment of 2017 (ZFDA, 2013) Policies, Regulations, guidelines, procedures, and reports of assessments done by the Tanzania Food and Drugs Authority (TFDA), now the Tanzania Medicines and Medical Devices Authority (TMDA) in 8th–9th February 2007, followed by East Africa Community (EAC) on 30th–31st July 2009, WHO World Bank on 11th October 2011 (Group, 2017), NEPAD Agency (*NEPAD Home | AUDA-NEPAD*, 2022) on 9th March 2016 and WHO Headquarter on 14th–15th May 2018 (WHO, 2018). In addition to the assessment reports, the selection of other regulatory documents was guided by predefined criteria that considered their relevance in evaluating the evolution of Zanzibar’s medicine regulatory affairs, publication date, and source credibility.

## Data collection tools

A review checklist was developed and used for document review. This study focuses on relevant regulatory documents. Additionally, the World Health Organization’s Global Benchmarking Tool (GBT) (World Health Organization, 2021) was used to provide standardised data collection and evaluation indicators.

## Data collection procedure

Documents were systematically reviewed by trained researchers with expertise in medicine regulatory affairs and public health. A standardised data extraction checklist was used to systematically record relevant information from the documents, ensuring consistency and comparability across the reviewed materials. Two researchers were paired to collect and review the documents independently, ensuring thoroughness and reducing bias. After the initial review, the pairs cross-checked their findings to identify any discrepancies or areas that required further clarification. Subsequently, a final reviewer reviewed the extracted data, consolidating the findings and ensuring the accuracy and completeness of the information.

## Data analysis

The collected data were examined and interpreted to uncover patterns, themes, and insights related to evaluation of the evolution of Zanzibar’s medicine regulatory affairs over the past 20 years. Data were first cleaned and formatted to ensure consistency, followed by identification of concepts and patterns. Regulatory System indicators from the WHO GBT were used, and the findings were interpreted to understand their significance and implications concerning the evolution of Zanzibar’s medicine regulatory affairs. Observational data were used to triangulate the findings and enhance their credibility.

## Ethical considerations

The study received ethical approval from the Zanzibar Health Research Ethics Committee (ZAHREC), with reference number ZAHREC/01/PR/AUG/2024/25. Additionally, a formal approval letter was issued by the Executive Director of the ZFDA before the commencement of the study. Confidential information was provided to the research team based on valid and sound requests. These requests were assessed by the Agency’s legal team to ensure their legitimacy. All researchers signed confidentiality and non-disclosure agreements prior to starting the review to safeguard the protection of confidential information.

## Results

The review of relevant regulatory documents provided the following findings, as the critical areas of assessment guided them in 2007, 2009, 2011, 2016, and 2018, and the actions taken were validated through observation of the physical verifiers.

## Legal provisions and guidelines

Assessed documents reported the existence of the Act for the Establishment of the ZFDB, Zanzibar Food, Drugs and Cosmetics (ZFDC) Act, 2006 and, later, the ZFDA, Zanzibar Food, Drugs and Cosmetics Act, 2017 (ZFDA, 2013). However, the Act did not establish the secretariat of the ZFDA, except for registration, which served as the secretariat to the Board of directors. In addition, the Act lacks provisions for the designation and appointment of analysts. In addition to medicine regulatory activities, the agency regulates the pharmacy profession in Zanzibar. Marked as a major gap, recommendations were made to revise the Act to establish the ZFDA.

In response to these recommendations, ZFDC Act No. 2 of 2006 (ZFDA, 2013) was revised and amended to provide for the missing power of appointments. The amendments included provisions for pharmacovigilance and post-marketing surveillance (PMS). ZFDA has implemented two PMS programs (2018/2021 and 2021/2024) and is currently reviewing its third program. Additionally, an electronic system for capturing and recording quality and safety issues, including adverse drug reactions, has been established (ZFDA, 2018a). The agency is also a full member of the WHO Programme for International Drug Monitoring (PIDM) (World Health Organization, 2022). Furthermore, a draft bill to establish a Pharmacy Council was prepared, pending governmental procedures for approval. The pharmacy council will eventually take over the regulation of the pharmacy profession in Zanzibar. However, until the council is established, the regulation of the pharmacy profession remains within the scope of work of ZFDA.

## Organization and good governance

Until the time of assessment, the agency did not have any official organizational structure. It is recommended that the agency consults with the TMDA for a model organisational structure. We found that ZFDA has an official organisational structure approved by the Civil Service Commission of the Revolutionary Government of Zanzibar (RGoZ) (Zanzibar, 2022).

## Strategic plan with clarified objectives

Until the 2011 assessment, the agency did not have a developmental strategy. The agency was recommended to develop a comprehensive five-year strategic plan covering all regulated products: Food, Drug, Cosmetics, and Medical devices. This assessment revealed that the latest strategic plan is dated 2014/2019; revisions are ongoing.

## Regulatory system

Previous assessments have reported the existence of a regulatory framework for medicine registration through the ZFDC Act. However, several gaps were identified, including the absence of regulations for medicine registration, a non-comprehensive list of guidance documents covering only certain types of pharmaceutical products, and the unavailability of mechanisms to ensure confidentiality and a code of conduct for internal and external experts. Furthermore, the assessment reported the non-existence of the requirement for holders of marketing authorisation of new medicinal entities to monitor adverse reactions actively and periodically report the results to the agency.

The assessments pointed out recommendations, including the need to consider developing regulations for the registration of medicinal products, updating existing guidelines for the registration of medicines, and having a mechanism for monitoring conflicts of interest, including the declaration of interest. Additionally, the assessment recommended the establishment of a mechanism to ensure confidentiality and a code of conduct for internal and external experts, as well as the provision of a requirement for holders of marketing authorisation for new medicinal products to monitor adverse reactions actively. The current observation revealed the agency’s active participation in regional and international colllaborations such as the East African Community (EAC) Medicine Registration Harmonization and the adoption of harmonised guidelines for medicine registration. This was further evidenced by the existance of a framework for good reliance practice (ZFDA, 2018b).

## Quality Management Systems (QMS)

As of the 2011 assessment, the agency had not established an operationalised quality management system. It was then recommended that the agency develop and institute a Quality Management System to improve organisational performance and customer satisfaction continually. The current state of the agency has revealed the existence of a fully functional and operational Quality Management System, justified by the attainment of ISO 9001:2015 and its accreditation on 16th January 2018.

## Human resource for regulatory activities

During 2011, the ZFDB had a relatively small number of staff to carry out regulatory activities, with only three pharmacists, one pharmaceutical technician, and four pharmaceutical assistants employed at headquarters in Unguja. At the seaport and airport, there were reported to be only 12 pharmaceutical assistants designated as drug inspectors who were working full-time. Pemba Suboffice had only two pharmacists, one pharmaceutical technician, and three drug inspectors. The assessment noted that the Board appointed and gazetted two legal officers from the Ministry of Health (MoH) to support the agency on legal matters.

Several gaps were raised, including an absence of structured provision of duties and responsibilities for regulatory staff, the unavailability of an official training programme for newly recruited staff, and the lack of a written code of conduct for staff involved in drug regulation. Despite having a staff appraisal system, it was found to be based on a confidential appraisal mechanism by supervisors whose reports were submitted to supervisors in the Ministry of Health (MoH) for confirmation. Furthermore, the agency lacked a systematic approach to identifying training needs, along with the lack of budgetary provision for staff training allocated by the agency. The gaps were accordingly recommended for their fulfilment, with a greater focus on establishing a staff remuneration package that will incentivize workers, prevent public-to-private sector migration, and minimise the potential for corruption.

Observations of the current state have revealed that the agency has developed job descriptions for each staff member, and a scheme of service for government agencies (Reference number IKL/TS/WA-12D/Vol.6/16) that promotes the benefits of the staff has been endorsed. However, this scheme is not specific to positions within an agency. Along with this, ZFDA hass developed a human resource development plan which outlines strategies for staff attraction and retention, including continuous professional development and a supportive work environment.

## Financial resources for regulatory activities

Among the outlined advantages, the agency was reported to have a legal mandate to collect fees from various services, including medicine registration, regulatory inspections, market control, premises registration, and licensing. However, the charges were minimal to enable effective medicine regulation. The fees were not legally defined in the regulations, and the financial support provided by the government was generally limited, rendering the implementation of medical regulation activities inefficient. It was then recommended that the current fees be revised to match other regional Medicine Regulatory Authorities (MRAs), and fees should be charged at a level that adequately reflects the actual cost of the medicine regulatory services.

The agency scheduled a periodic review of fee regulations to circumvent financial resource limitations and adhere to recommendations. Addressing this issue at the central level, the Government of Zanzibar allows ZFDA to retain its collections (relevant fees and charges), thereby decentralising financial resources and enhancing the agency’s capacity to manage its operations smoothly, and moreover the agency received a a government subvention to the agency (Reference number BC.82/300/01/10/135) was initiated in the 2015/16 financial year. In addition, the agency benefits from the financial support provided by various stakeholders and development partners.

## Infrastructure and equipment

The agency reported an acute shortage of workspace for staff with limited equipment to sustain regulatory activities. Three computers with no information technology (IT) systems and one vehicle were available for administrative and inspection activities. The assessment then recommended that the government consider constructing offices that would accommodate all staff involved in medicine regulatory functions and provide the agency with vehicles to assist in administrative and inspection activities. The agency also recommended a comprehensive Management Information System (MIS) to link all key regulatory activities through a network.

Current observations and reviews have found that the agency has been relocated to a new office building and state-of-the-art laboratory built on the Unguja ZFDA premises. Cumulatively, the office spaces available can accommodate approximately 149 staff. Equipment capacity increased with the procurement and transportation of several IT accessories. The agency has eight vehicles and two motorcycles to support administrative and inspection activities.

## Mechanisms for transparency, accountability, and communication

The assessment reported that the agency’s information on legislation and regulations was publicly available and regularly updated in hard copies. However, several regulations, guidelines, and standard operating procedures have yet to be developed. Until the assessment, the agency had not published any obligatory information, such as annual reports. Information on authorised products and licensed companies is publicly available in hard copies, excluding Supplementary Protection Certificates (SPCs). The agency was reported to work closely with non-governmental organisations representing health professionals, industries, consumers, and patients to develop regulations and guidelines. It was also realised that the Act provides an appeal mechanism.

The major gaps identified were the decision-making criteria and processes not being documented; however, the final decision was publicly available. Moreover, a documented confidentiality policy is unavailable to the agency. The identified gaps are recommended for action. A recommendation was also made for the agency to consider developing a website to publish the various decisions made by the agency.

The present observational checklist marks the existence of the agency website (Zanzibar Food and Drug Agency, 2024), where regulatory decisions are made available to the public.

## Discussion

This study examines the transformation of Zanzibar’s medicine regulatory affairs over the past two decades. Through an analysis of the evolution of the regulatory system, valuable insights were gained into the changes that have occurred, challenges encountered, and achievements made in ensuring the safety, efficacy, and quality of medicine in Zanzibar.

## Historical overview

Over the past two decades, Zanzibar has witnessed significant developments in its medicine regulatory affairs. Initially, the regulatory landscape was characterised by limited legal provisions, a lack of organisational structure, and inadequate resources. These challenges have been documented to limit regulators’ efforts to protect and advance public health by pursuing two main goals: ensuring drug safety, quality, and efficacy, and facilitating access to medically useful drugs (Olson, 2014). However, recognizing the importance of a robust regulatory framework, efforts have been made to address these challenges and improve the system.

## Legal provisions and regulations

One of the key transformations in Zanzibar’s medicine regulatory affairs has been strengthening of legal provisions and regulations. In the early stages, the act did not provide for establishing a secretariat or the designation and appointment of analysts. As the main operator, the secretariat plays a crucial role in supporting the overall functioning and operation of the National Regulatory Authority/Agency (NRA). The appointment power vested in the NRA ensures that it can effectively control and regulate the pharmaceutical market (Ndomondo-Sigonda et al., 2017). Legislative amendments have addressed these shortcomings and enhanced regulatory frameworks. This underscores the legal mandates granted to the ZFDA as Zanzibar’s NRA, significantly strengthening its capacity to regulate and effectively oversee the pharmaceutical sector.

The development of regulations for registering medicinal products, updating guidelines, and implementing mechanisms to manage conflicts of interest and confidentiality are important milestones achieved during this transformation. These regulatory advancements have played a crucial role in ensuring adherence to international standards and best practices in medicine regulation. The ZFDA has strengthened its regulatory function in the inspection by joining the medicine regulatory harmonisation initiative spearheaded by the East African Community (Ndomondo-Sigonda et al., 2021).

The development of regulations for registering medicinal products and medical devices, updating guidelines, and implementing mechanisms to manage conflicts of interest and confidentiality are important milestones achieved during this transformation. The ongoing initiatives to advancing the agency’s capabilities in key areas such as post-marketing surveillance (PMS) and pharmacovigilance (PV) reflects a commitment to progress toward a higher maturity level in regulatory practices. These regulatory advancements have played a crucial role in ensuring adherence to international standards and best practices in medicine regulation. The ZFDA has strengthened its regulatory inspection function by joining the medicine regulatory harmonisation initiative spearheaded by the East African Community (Ndomondo-Sigonda et al., 2021).

Through these collaborative efforts, the ZFDA has enhanced its inspection capabilities, ensuring that medicinal products meet stringent safety, quality, and efficacy standards. This alignment with regional harmonisation initiatives has further solidified Zanzibar’s commitment to maintaining a robust and effective medicine regulatory framework.

## Organisational development, planning and good governance

Another significant aspect of the transformation process is developing an organisational structure and promoting good governance within the regulatory system. Independent, efficient, and unbiased decision-making requires a comprehensive organisational structure and principles of good governance, including accountability and transparency, to be in place (World Health Organization, 2016). Initially, the organisational structure was poorly defined, leading to inefficiencies and a lack of coordination. However, efforts have been made to establish and approve a model organisational structure (Zanzibar Food and Drug Agency, 2024), drawing inspiration from similar organisations such as the Tanzanian Medicines and Medical Devices Authority (Tanzania Medicines and Medical Devices Authority (TMDA), 2024).

The ZFDA has enhanced its operational efficiency and effectiveness by implementing a well-defined organisational structure. The new structure supports clear roles and responsibilities, improves department communication and coordination, and promotes accountability and transparency in regulatory decision-making. This alignment with good governance practices is crucial for building a robust and reliable regulatory authority capable of safeguarding public health by effectively regulating medicines.

Furthermore, introducing a strategic plan with clarified objectives has been instrumental in providing a roadmap for the agency’s activities and aligning them with long-term goals. Developing a five-year strategic plan covering all regulated products laid the foundation for effective planning, resource allocation, and continuous improvement within the regulatory system. As with any properly functioning NRA, a strategic plan provides a clear sense of direction, a well-defined approach, and effective execution of plans that will foster the success of the NRA (Pejović et al., 2016).

This strategic plan has enabled the ZFDA to set measurable goals, prioritise key regulatory activities, and allocate resources efficiently. It has also facilitated the monitoring and evaluating of progress, ensuring that the agency remains focused on protecting public health. By systematically addressing gaps and challenges, the strategic plan has driven continuous improvement and innovation within the regulatory framework, reinforcing the ZFDA’s commitment to upholding the highest standards in medicine regulation.

## Medicine regulatory system

The transformation of Zanzibar’s medicine regulatory affairs also strengthened its regulatory systems. Initially, there were limitations regarding skilled human resources, monitoring adverse reactions, managing conflicts of interest, and providing adequate staff training. These gaps pose challenges to the effective regulation of medicines.

To address these issues, this study recommends implementing mechanisms for monitoring conflicts of interest and establishing a formal or structured training programme for staff. These measures are crucial for enhancing regulatory capacity and ensuring the competence of regulatory personnel.

The agency engages with various reliance mechanisms both internationally and regionally. ZFDA has a framework for reliance practices that includes recognising decisions from Stringent Regulatory Authorities (SRAs), WHO prequalification, and the East African Community (EAC) harmonization programme. Medicines and medical devices approved by these entities undergo an abridged registration process in Zanzibar, thus streamlining regulatory procedures while preserving standards and autonomy. Additionally, URT has ratified the African Medicine Agency (AMA) Treaty and deposited the instrument of ratification (Bevin Likuyani, 2023). The ZFDA anticipates benefiting from the AMA’s work products and reliance mechanisms once the agency becomes fully operational.

## Resource allocation and infrastructure

A significant aspect of the transformation process is allocating financial resources and improving infrastructure to support regulatory activities. Initially, there was limited government financial support for medicine regulation, resulting in insufficient funds for effective regulatory activities (Group, 2017). Additionally, addressing infrastructure limitations such as the shortage of workspace, limited IT systems and accessories, and inadequate vehicles is crucial for the smooth functioning of regulatory agencies. Constructing offices, acquiring necessary equipment, and developing a comprehensive Management Information System (MIS) are essential to strengthen infrastructure and enhance operational efficiency (Ndomondo-Sigonda et al., 2017).

Despite the challenges associated with small island context, ZFDA maintains a strong technical capacity. The agency ensures that its staff receive comprehensive training through on-the-job experiences, long-term courses, and short-term workshops. ZFDA leverages harmonisation efforts and partnerships with universities, research and development labs, and organisations such as WHO, EAC, and AUDA-NEPAD to provide its staff with essential training programmes. These measures enable ZFDA to build and sustain the technical capacity necessary to operate effectively and progress towards a maturity level comparable to the mainland Tanzania Medicines and Medical Devices Authority (TMDA).

The agency has established a state-of-the-art laboratory facility and modern office as part of its revolutionary infrastructural development (*UFUNGUZI WA MAABARA MPYA NA YA KISASA YA ZFDA WILAYA YA MAGHARIBI “B”- MOMBASA, ZANZIBAR. – ZANZIBAR FOOD AND DRUG AGENCY*, 2023). The new premises accommodate up to 30 technical and 20 non-technical personnel with nine offices, including those for food sampling and research and development, food chemistry, microbiology, quality and technical advisory, and medicine and cosmetics analysis. The laboratory is equipped with analytical equipment such as a tandem gas chromatography-mass spectrometer (GC-MS/MS), Kjeldahl Analyzer, High-Performance Liquid Chromatography (HPLC), and Matrix-Assisted Laser Desorption/Ionization-Time of Flight (MALDI-ToF).

## Lessons learned and future directions

The evolution of Zanzibar’s medicine regulation over the past two decades provides valuable lessons for other regulatory systems facing similar challenges, especially in LMICs. This transformation underscores the importance of continuous improvement, strategic planning, resource allocation, and stakeholder engagement in building an effective and efficient regulatory framework.

The Agency strives to sustain progress and strengthen its regulatory systems by regularly monitoring regulatory performance and output, fostering transparency, accountability, and communication, and embracing technological advancements to enhance regulatory efficiency.

Collaboration with regional and international regulatory bodies, such as the East African Community Medicines Regulatory Harmonization (EAC-MRH) (Arik et al., 2020) and African Medicine Regulatory Harmonization (AMRH) (Mwangi, 2016; Ndomondo-Sigonda & Ambali, 2011), contributes to harmonising regulatory practices, sharing best practices, and ensuring the availability of safe and high-quality medicines in the region.

This study opens several avenues for future research in the field of medicine regulatory affairs in Zanzibar. First, further investigation should be conducted to assess the impact of regulatory transformations on key outcomes, such as the quality and availability of medicines, patient safety, and healthcare outcomes. Such studies can provide evidence for the effectiveness of interventions and inform continuous improvement efforts.

Additionally, research focusing on stakeholder perspectives, including healthcare professionals, consumers, and industry representatives, could provide valuable insights into their experiences and perceptions of the regulatory system. Understanding the viewpoints and expectations of different stakeholders can help develop more inclusive and responsive regulatory frameworks.

Furthermore, comparative studies across countries or regions in similar contexts can provide insights into the best practices and lessons learned from diverse regulatory systems. Such comparative analyses can facilitate knowledge exchange, foster collaboration, and support evidence-based policymaking.

## Limitations

Although this study offers valuable insights, it is essential to acknowledge its limitations. Primarily, its reliance on retrospective analysis of assessment reports and current status observations may introduce bias and overlook the complex distinctions of the transformation process. To mitigate this, future research could integrate qualitative interviews and surveys to capture stakeholder perspectives on Zanzibar’s regulatory landscape.

Moreover, while our findings may offer insights applicable across various settings, they are particularly relevant in resource-constrained environments. Further research should explore the unique challenges and opportunities in different contexts to provide a more comprehensive understanding of the transformation process in medicine regulations.

## Conclusion and recommendations

Over the past two decades, Zanzibar’s medicine regulations have evolved significantly, and resulting in notable achievements and improvements in the regulatory framework. Strengthening legal provisions, organisational development, strategic planning, and resource allocation has enhanced regulatory capacity and ensured the safety, efficacy, and quality of medicines.

However, challenges remain, and the ZFDA must continue to address these issues to improve its regulatory system. By learning from past experiences, embracing innovation, and fostering collaboration, Zanzibar can further evolve its medicine regulations and safeguard public health.

## Data Availability

Data supporting the findings of this study are available from the corresponding author upon reasonable request.
